# Spermidine enhances chilling tolerance of kale seeds by modulating ROS and phytohormone metabolism

**DOI:** 10.1371/journal.pone.0289563

**Published:** 2023-08-03

**Authors:** Dongdong Cao, Yutao Huang, Gaofu Mei, Sheng Zhang, Huaping Wu, Tiyuan Zhao

**Affiliations:** 1 Institute of Crop and Nuclear Technology Utilization, Zhejiang Academy of Agricultural Sciences, Hangzhou, China; 2 Taizhou Agricultural Technology Extension Center, Taizhou, China; 3 Huzhou Keao Seed Co., Ltd., Huzhou, China; Bangabandhu Sheikh Mujibur Rahman Agricultural University, BANGLADESH

## Abstract

Chilling stress is an important constraint for kale seed germination and seedlings establishment. It is vital to develop an effective approach to enhance kale seed germination ability under chilling stress. The present study reported that spermidine (Spd) could improve seed chilling tolerance in two kale cultivars ‘Nagoya’ (MGW) and ‘Pigeon’ (BB) during germination. The results showed that MGW was cold tolerant with a 90.67% germination percentage (GP) under chilling stress, while BB was cold sensitive with a 70.67% GP under chilling stress. Spd content in MGW and BB seeds during seed germination were up-regulated and down-regulated by chilling stress, respectively. Besides, chilling stress apparently decreased the gibberellin (GA) and ethylene (ET) contents, while increased the levels of abscisic acid (ABA) and reactive oxygen species (ROS) in MGW and BB seeds during germination. Exogenous Spd application increased GA, ET contents and decreased ABA content through regulating the gene expressions of metabolic-related enzymes, thus effectively alleviating the low temperature damage on kale seed germination. Besides, Spd significantly increased the activities of superoxide dismutase (SOD) and peroxidase (POD), and reduced the levels of hydrogen peroxide (H_2_O_2_) and superoxide anion (O_2_·^-^). The present study demonstrated that endogenous Spd metabolism plays an important role in kale seed germination under chilling stress. The effect of exogenous Spd on the metabolism of endogenous Spd, GA, ABA, ET and antioxidant enzymes might be the important reason for promoting the kale seed vigor at low temperature.

## 1 Introduction

Chilling stress is a major abiotic constraint that impacts the seed germination and plant growth [1]. Kale (*Brassica oleracea* var. *Acephala* L.) belongs to species *Brassica Oleracea*, family *Brassicaceae*, is an important economic crop, which has come under the spotlight in recent years due to its high ornamental and edible values [2]. Kale plant exhibits strong cold resistance during plants growth period, and a low temperature at 0 °C~3 °C is an important condition for plant leaves color change. However, the kale seed germination and seedlings establishment are greatly affected by low temperature damage, which remains a serious problem encountered in the production of kale [3]. It is of great significance to explore the innovative methodologies for the improvement of low temperature tolerance of kale seed and seedlings.

Polyamines (PAs), mainly including putrescine (Put), spermidine (Spd) and spermine (Spm), are a class of cationic compounds which are considered as important modulators involving in plant growth and development regulation [4–6]. The biosynthesis of polyamines in higher plant has been extensively investigated, which involves five key synthetases, including arginine decarboxylase (ADC), ornithine decarboxylase (ODC), S-adenosylmethionine decarboxylase (SAMDC), spermidine synthase (SPDS) and spermine synthase (SPMS) [7]. Spd was proved be closely involved in the plant response to abiotic stress. Spd could mediate multiple plant defense responses through different signal molecules like hydrogen peroxide (H_2_O_2_) and nitric oxide, thus responding to low temperature stress [8, 9]. Overexpression of low temperature responsive transcription factor *MfERF1* could promote the Spd synthetic-genes expressions and the conversion of polyamines, subsequently enhancing the cold tolerance of tomato plants [10]. Moreover, overexpression of *slSAMDC* induced the expression and accumulation of pathology-related protein PR1b1 and boosted the resistance to chilling stress in tomato under chilling stress [11]. However, there were little reports on the role of Spd in regulating seed germination. *OsSPMS1* participates in polyamine and ethylene homeostasis and negatively regulated seed germination and plant growth in rice [12]. It was reported that exogenous Spd enhanced rice seed germination at low temperature by regulating antioxidant enzyme system and photosynthetic function [13]. However, the mechanisms of Spd involved in seed germination under low temperature remain elusive, especially in Kale.

The dynamic balance of synthesis and catabolism of gibberellin (GA) and abscisic acid (ABA) plays an important role in plant seed dormance and germination [14]. Seed dormance was generally mantiand by high ABA content and broken by GA accumulation in many species [15–17]. GA 20-oxidase (GA20ox) and GA3-oxidase (GA3ox) were the main enzymes involved in bioactive GA biosynthesis and related-genes were highly expressed at the early stage of seed germination, which induced the excessive accumulation of GA in the seed embryo [18]. While GA 2-oxidase (GA2ox) was known as the key enzyme in GA catabolism. Chilling stress delayed *Arabidopsis* seed germination process through suppressing the expression of *GA3ox1* and *GA3ox2* [19]. 9-cis-epoxycarotenoiddioxygenase (NCED) was the key enzyme involved in ABA biosynthesis, while *CYP707A* encoding abscisic acid 8-hydroxylase (ABA8ox) as a key gene for ABA catabolism [14]. In the *cyp707a2* deletion mutant, both GA content and *GA3ox1* expression significantly decreased, indicating that ABA negatively regulated GA synthesis to restrain *Arabidopsis* seed germination. Similarly, GA also inhibited the ABA synthetic route, and the antagonism of ABA and GA was mainly regulated by the Ap2 type transcription factor [20].

Ethylene (ET) is another important phytohormone which closely involved in fruit maturation, seed germination and palnt response to abiotic stress [21]. 1-Amicocyclopropane-1-carboxillic-acid (ACC) was the direct precursor for ethylene synthesis, which was synthesized from S-adenosyl-Met by ACC synthase (ACS). ACC oxidase (ACO) catalyzes the last step of ET biosynthesis from ACC [22]. The production rate of ET increased significantly during seed germination in rice, soybean, corn and wheat under salinity and chilling stress [23]. Kozarewa et al. reported that exogenous ACC or ET treatment could alleviated the thermo-dormancy of lettuce seeds [24]. Moreover, ET application could alleviate the inhibitory effects of ABA on plant seed germination [25, 26].

This study aimed to elucidate the further mechanism of Spd promoting kale seed germination under chilling stress. Two kale cultivars ‘Arbequina’ (MGW) and ‘Picual’ (BB), which differ in seed chilling tolerance, were used as experimental materials. The effects of Spd treatment on antioxidant enzymes activities, phytohormones (PAs, GA, ABA, ET) contents and corresponding-genes expressions during germination were test. The present study has practical value for better understanding on the potential mechanism of Spd in kale seed chilling tolerance enhancement.

## 2 Materials and methods

### 2.1 Experimental materials

Two kale (*Brassica oleracea* var. *acephala* L.) cultivars, MGW and BB, were chosen according to their chilling stress tolerance during germination, low and high, respectively. Kale seeds were obtained from the Zhejiang academy of agricultural sciences. Spermidine was purchased from Shanghai Aladdin Reagent Co., Ltd.

### 2.2 Seed treatment and seed germination test

Six seed treatments were performed in the present study (Table 1). Kale seeds were immersed in 0.5 mM Spd solution and purified water at the ratio of 1:15 (m/V) for 12 h in the dark at 25 °C. The priming concentration of Spd was determined by preliminary experiments. During the priming period, ventilation was constantly maintained by virtue of the air pump. After the initiation, seeds were taken out, washed with purified water for three times immediately and floated with absorbent paper. Subsequently, the seeds were dried to original water content at room temperature.

**Table 1:** Seed treatments performed in the study.

Treatments	Variety	Priming treatment	Seed germination temperature (°C)
MGW + Cn	MGW	Purified water	25
MGW + Cs	MGW	Purified water	25
MGW + Cs + Spd	MGW	0.5 mM Spd solution	13
BB + Cn	BB	Purified water	25
BB + Cs	BB	Purified water	25
BB + Cs + Spd	BB	0.5 mM Spd solution	13

MGW: kale variety ‘Nagoya’; BB: kale variety ‘Pigeon’.

Fifty kale seeds were germinated in each germination boxes (12 × 12 × 6 cm, length width height size) containing 3 layers of moistened filter paper, and four duplicates were set. Subsequently, the germination boxes were incubated under a diurnal cycle of 8 h of light and 16 h of darkness at 25°C and 13°C, respectively. Seeds were considered as germination when the radicle reached 2 mm. The geminated seeds numbers were counted daily. Then, the germination energy (GE) and germination percentage (GP) were determined on day 3 and day 7, respectively. After growing for 7 d, ten seedlings of kale randomly selected from each replicate were used to measure seedlings qualities. The seedling height was manually detected with a vernier caliper. The kale seedlings were dried at 80°C for 24 h, and followed by the detection of seedling dry weight. The germination index (GI) was measured based on the formula GI = ∑(Gt/Tt), where Gt corresponds to the germinated seeds number on the t day; Tt is the time corresponding to Gt in days. Seed vigor index (VI) was measured based on the formula VI = GI × seedling dry weight.

### 2.3 Determination of PAs, GA, ABA and ET contents

PAs determination from seeds was performed by high performance liquid chromatography (HPLC) [27]. Ten μL samples were injected into a 6.0 mm × 150 mm, 5 mm particle size reverse-phase (C18) column (Shim-Pack CLC-ODS) and eluted with 64/36 (v/v) methanol/water at a flow rate of 0.8 mL·min^−1^.

GA and ABA extraction from kale seeds were performed by HPLC [27]. The HPLC system equipped with ultraviolet detector and a 6.0 mm × 120 mm, 5 mm particle size reverse-phase (C18) column (Shim-Pack CLC-ODS) was used for identification and calculation of ABA and GA content in the extracting solution. The mobile phase methanol/water (64:36, v/v) was run at a flow rate of 1.0 mL·min^-1^.

The ET production rate by kale seeds was determinate with the method of Zhang et al. with small changes [28]. Thirty kale seeds of each treatment were enclosed in 10 mL of air-tight container for 3 h at 25°C. Subsequently, 1 mL of the headspace gas was collected and injected into a gas chromatograph (model Agilent, 6890 N, USA) equipped with an activated alumina column and a flame ionization detector. The measurement conditions were as follows: chromatograph column, HP-55% phenyl methyl siloxane, 30 m capillary alumina column (Agilent 19091 J-413); the temperature of column and detector was 75 °C and 145 °C, respectively; the flow rate of carrier gas N_2_ was 45 mL·min^−1^ and the hydrogen pressure was 0.55 kg·cm^−2^.

### 2.4 H_2_O_2_ and superoxide anion (O_2_·^-^) analysis

H_2_O_2_ content was determined with the method of Li et al. [29]. The H_2_O_2_ content was measured according to the absorbance of supernatant at 390 nm. O_2_·^-^ content was determined using the method of Jiang and Zhang [30]. The O_2_·^-^ content was determined with the absorbance of supernatant at 530 nm.

### 2.5 Antioxidant enzymes

Seeds samples were homogenized with 8 mL of potassium phosphate buffer (50 mM, pH 7.8), followed by centrifuging at 12,000 × g for 20 min at 4 °C. The supernatant was collected and used for the measurement of superoxide dismutase (SOD), catalase (CAT) and peroxidase (POD) activities. The analysis of the activities of antioxidant enzymes in kale seeds was performed with the method of Qiu et al. [31].

### 2.6 Real-Time Quantitative PCR

The total RNA of kale seeds was extracted with the plant RNA extraction kit (Huayueyang, Shanghai, China). The RNA purity and concentration were detected by the NanoDrop 1000 spectrophotometer (NanoDrop Technologies, Wilmington, USA). RNA reverse transcription test was carried out through the PrimerScriptTM RT reagent Kit (Takara, Japan). The qPCR protocol was performed on a LightCycler 480 Real-Time PCR instrument (Roche) with the SYBR-Green PCR Master kit (Applied Biosystems, Foster City, CA, USA). Primers applied in this study were listed in S1 Table. The expression quantities of genes were calculated according to the 2-ΔΔCT method with *actin* as the internal reference gene. Real-time PCR analysis was performed with three biological replications, and each was made in three technical replicates. All data were expressed as the mean SD after normalization.

### 2.7 Statistical analyses

The statistical analysis was carried out using one-way analysis of variance (ANOVA) on Statistical Analysis System (SAS) software. The means testing was performed with the least significant difference at the *P ≤* 0.05 level (Lsd_0.05_). Prior to statistic and statistical comparison, the percentage data were transformed in accordance with y = arcsin [sqrt (x/100)].

## 3 Results

### 3.1 Effects of exogenous Spd on kale seed germination and seedling growth under chilling stress

**Fig 1:**
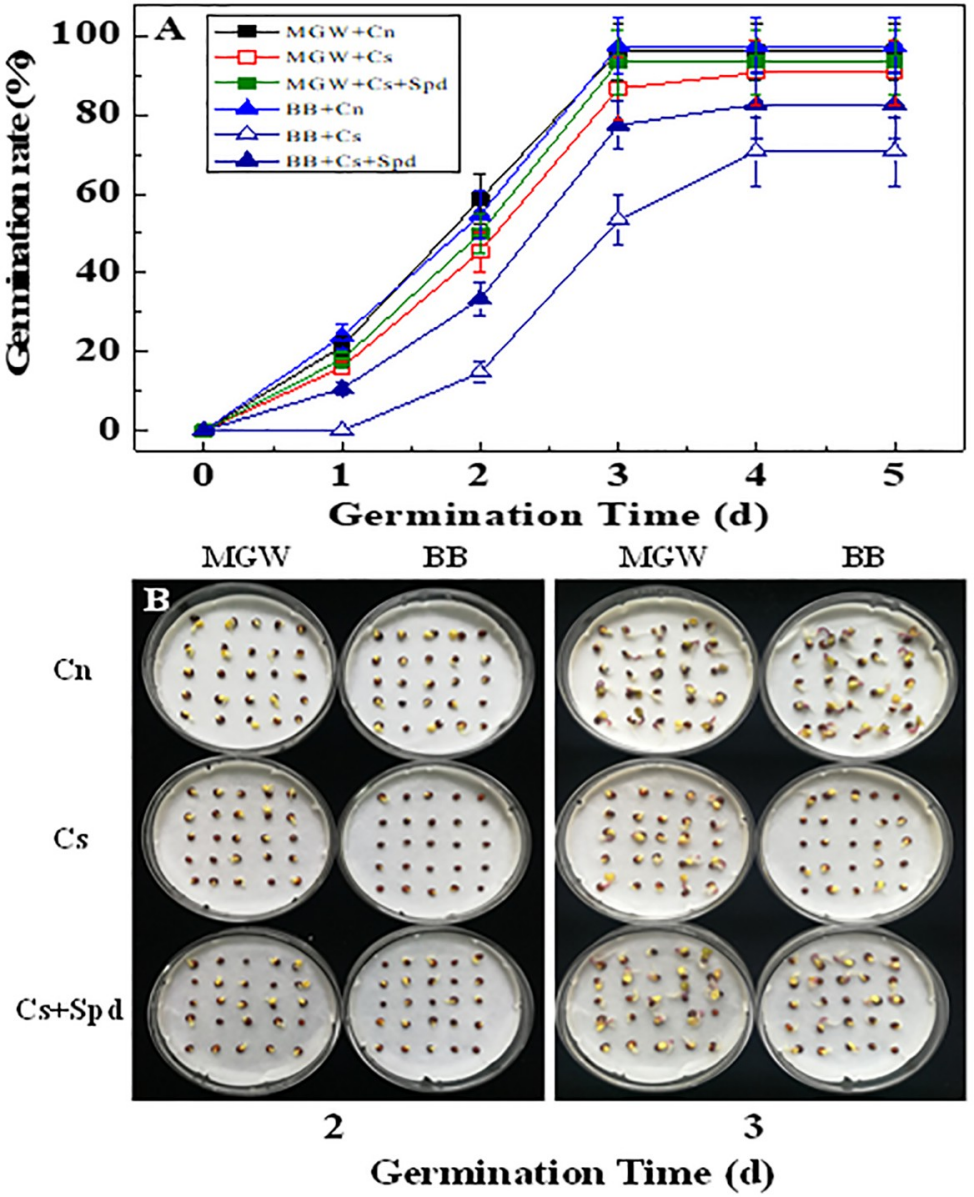
Effect of exogenous Spd on kale seeds germination under chilling stress. A: Time courses of kale seeds germination; B: Seed germination of kale at 2, 3 d of germination time. MGW: kale variety ‘Nagoya’; BB: kale variety ‘Pigeon’; Cn: no chilling stress (25 °C) + distilled water; Cs: chilling stress (13 °C) + distilled water; Cs + Spd: chilling stress (13 °C) + spermidine.

Chilling stress significantly inhibited the seed germination of MGW and BB, while BB was more sensitive to low temperature (Fig 1). Chilling stress significantly reduced the GE, GP, GI and VI of both cultivars (Table 2). Noticeably, exogenous Spd significantly increased the GE, GP, GI, and VI in BB seeds under chilling stress; while no significant difference on GE, GP, GI, and VI were detected between MGW + Cs and MGW + Cs + Spd treatments (Table 2).

**Table 2:** Effect of exogenous Spd on germination energy (GE), germination percentage (GP), germination index (GI) and vigor index (VI) of kale seeds under chilling stress.

Treatment	GE (%)	GP (%)	GI	VI
MGW + Cn	96.00 ± 7.26a	96.00 ± 7.26a	13.11 ± 0.82a	0.49 ± 0.03a
MGW + Cs	86.67 ± 5.09b	90.67 ± 7.13b	11.36 ± 0.79b	0.34 ± 0.04b
MGW + Cs + Spd	87.33 ± 6.25b	92.00 ± 6.25b	11.92 ± 1.06b	0.37 ± 0.03b
BB + Cn	97.33 ± 6.31a	97.33 ± 6.31a	13.39 ± 0.48a	0.47 ± 0.02a
BB + Cs	53.33 ± 6.15d	70.67 ± 8.75d	6.14 ± 0.37d	0.16 ± 0.02d
BB + Cs + Spd	77.33 ± 6.07c	82.67 ± 6.54c	9.50 ± 0.44c	0.26 ± 0.03c

MGW: kale variety ‘Nagoya’; BB: kale variety ‘Pigeon’; Cn: no chilling stress (25 °C) + distilled water; Cs: chilling stress (13 °C) + distilled water; Cs + Spd: chilling stress (13 °C) + spermidine. *Values followed by a different letter within a column are significantly different at the 0.05 probability level.

**Fig 2:**
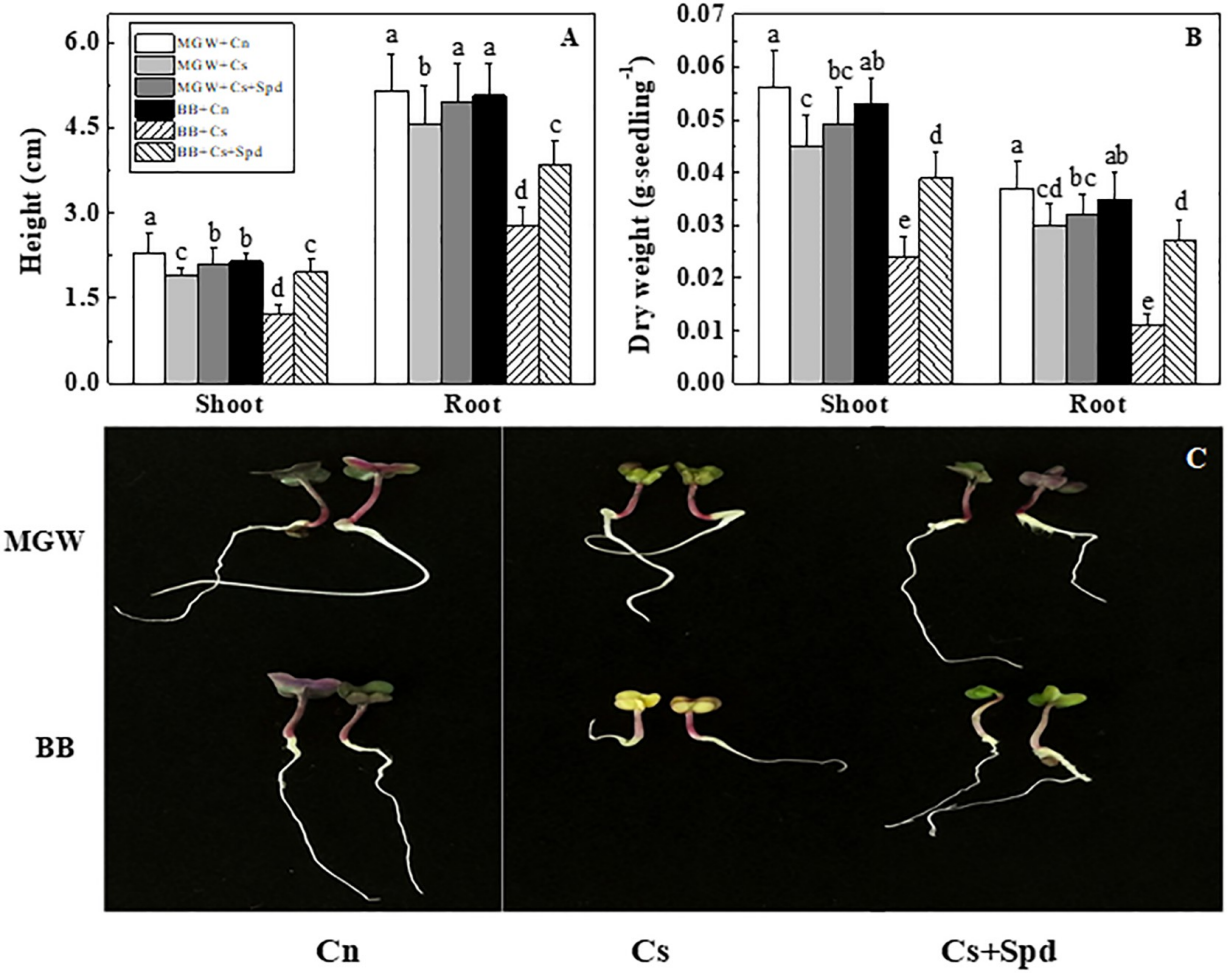
Effect of exogenous Spd on kale seedling characteristic at 5 d of germination time under chilling stress. A: The seedling height of different treatments under chilling stress; B: The seedling dry weight of different treatments under chilling stress; C: The seedling characteristic of different treatments under chilling stress. MWG, BB, Cn, Cs, Cs + Spd see Fig 1. Different small letters on top of the bars indicated significant differences (p ≤ 0.05, LSD) among treatments at same sown time.

Chilling stress significantly suppressed the seedling characteristic in both cultivars. On d 7^th^ of germination, the root length, shoot height, root dry weight and shoot dry weight of MGW + Cs decreased by 11.2%, 18.2%, 18.9% and 19.6% respectively, compared with MGW + Cn; while those of BB + Cs declined by 45.0%, 42.8%, 68.6% and 54.7%, separately compared with BB + Cn. Consistent with above findings, the seedling characteristics of BB under chilling stress were significantly lower than those in Spd treatment on d 7^th^ of germination (Fig 2). In addition, Spd treatment also enhanced the seedling height of MBG seedlings at low temperature, but made no significant difference to seedlings dry weight.

### 3.2 Effects of exogenous Spd on polyamines contents and related-gene expressions during kale seed germination under chilling stress

**Fig 3:**
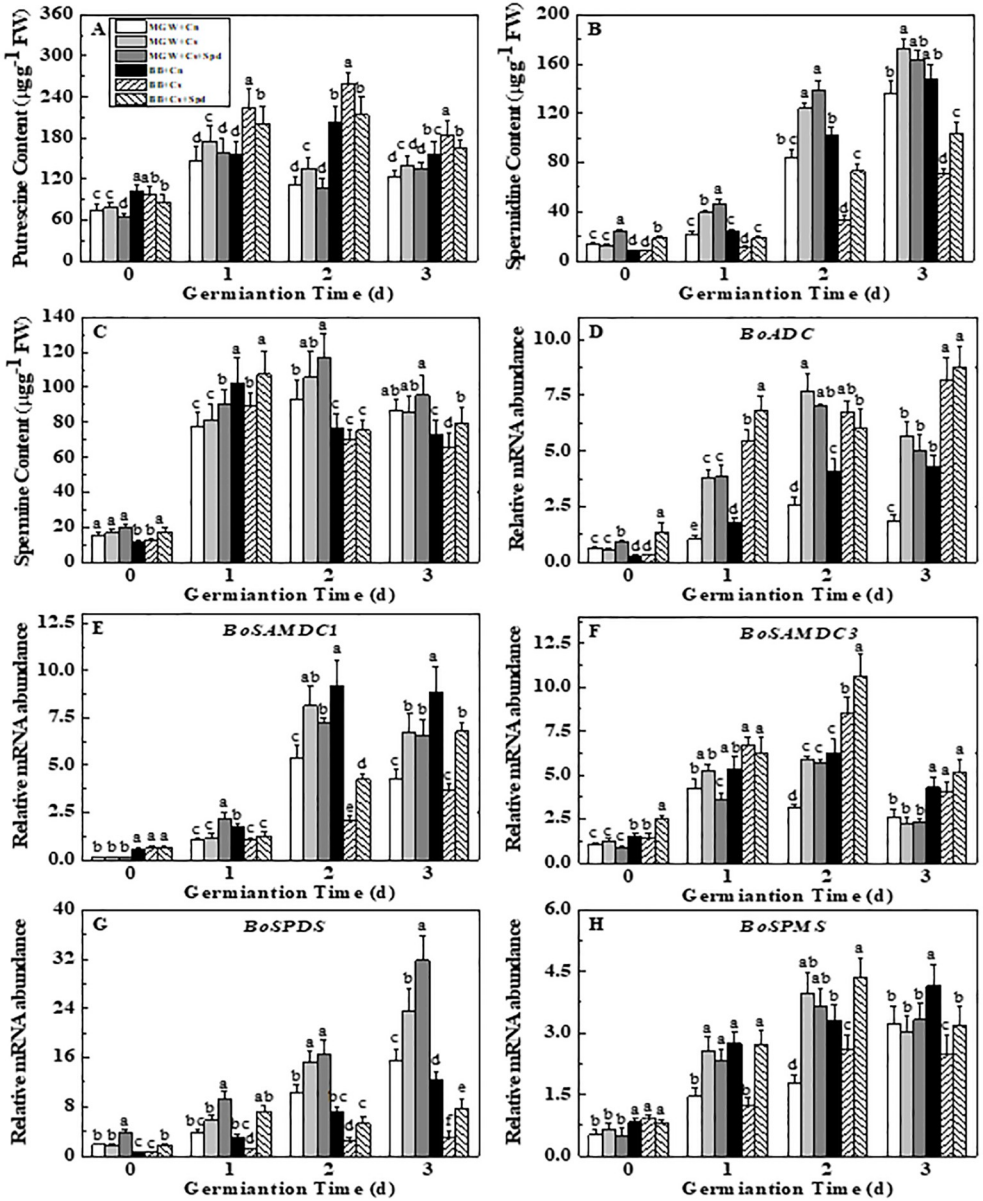
Effects of Spd on Putrescine (A), Spermidine (B), Spermine (C) contents and metabolism-related genes relative expressions (D-H) of kale seeds during germination time under chilling stress. ADC: arginine decarboxylase; SAMDC: s-adenosylmethionine decarb-oxylase; SPDS: spermidine synthase; SPMS: spermine synthase. MWG, BB, Cn, Cs, Cs + Spd see Fig 1. Different small letter(s) on top of the bars indicated significant differences (p ≤ 0.05, LSD) among treatments at same sown time.

Chilling stress affected the polyamines metabolism during the kale seed germination (Fig 3). Chilling stress significantly increased the Put content in both cultivars. The Spm content was found decreased in BB seeds with chilling stress. However, no significant change in Spm content was observed between MGW and MGW + Cs treatments. Noticeably, the response of Spd metabolism to chilling stress varied among cultivars. At low temperature, the Spd content in MGW outstandingly elevated, while it markedly declined in BB. Exogenous Spd increased the endogenous Spd content of BB on d 1^st^-3^rd^ of germination, and it had no significant effect on MGW under chilling stress. Moreover, exogenous Spd remarkably lowered the Put content in both cultivars on d 1st and 2nd of germination.

Chilling stress significantly increased the expressions of *BoADC* and *BoSAMDC1* in both MGW and BB seed at 2^nd^ and 3^rd^ d of germination (Fig 3D and 3E). However, the expressions of *BoSPDS* and *BoSPMS* in MGW were significantly up-regulated, while down-regulated by chilling stress in BB at 1^st^ and 2^nd^ d of germination (Fig 3G and 3H). Exogenous Spd up-regulated the *BoSAMDC1*, *BoSPDS* and *BoSPMS* expressions in BB on d 1^st^-3^rd^ of germination, as well as *BoSPDS* expression in MGW. However, no significant difference in *BoSAMDC1*, *BoSAMDC2* and *BoSPMS* expressions was detected between treatments of MGW + Cs and MGW + Cs + Spd during seed germination (Fig 3E, 3F and 3H).

### 3.3 Effects of exogenous Spd on GA content and related-gene expressions during kale seed germination under chilling stress

**Fig 4:**
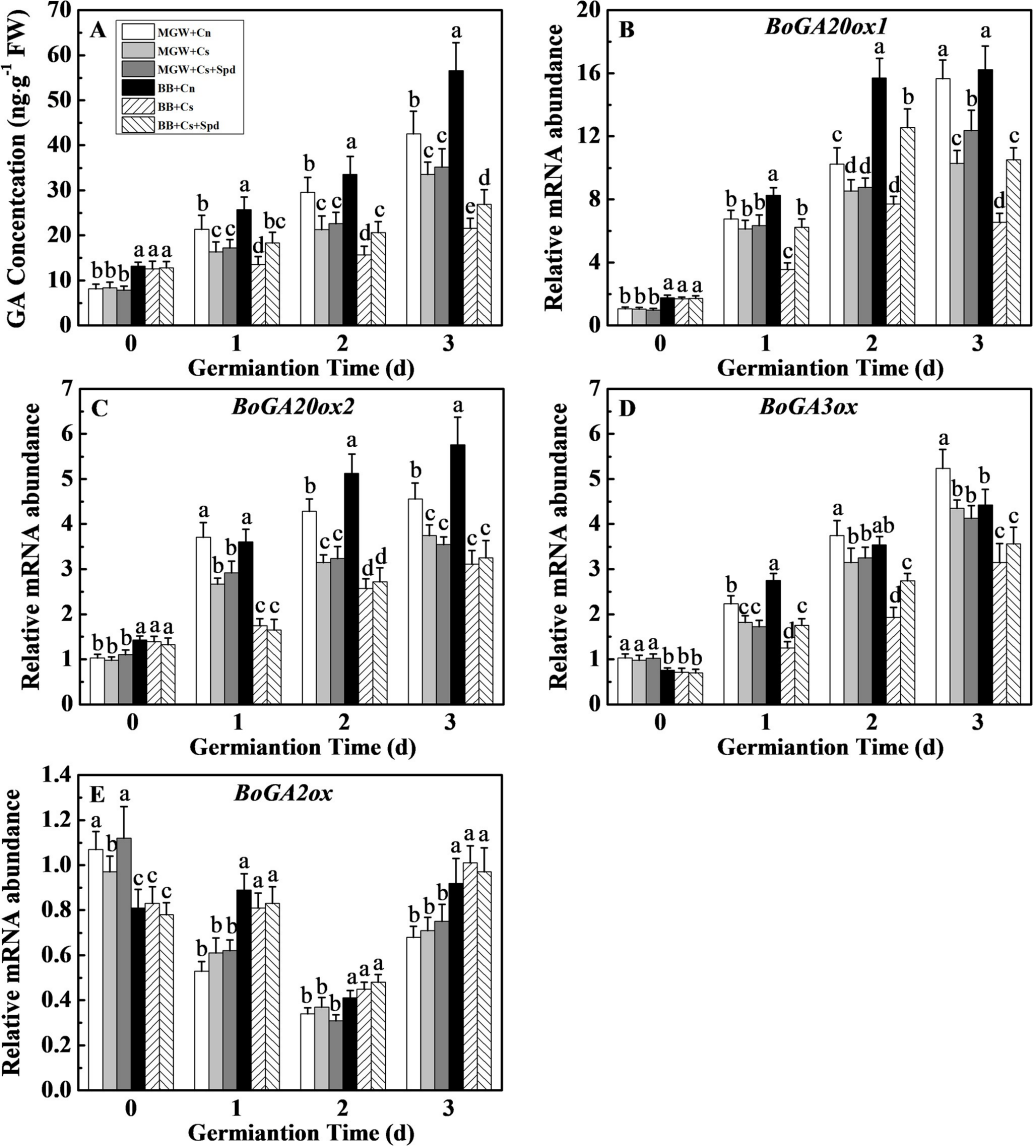
Effects of Spd on GA content (A) and relative expressions of *BoGA20ox1* (B), *BoGA20ox2* (C), *BoGA3ox* (D) and *BoGA2ox* (E) of kale seeds during germination time under chilling stress. ABA: abscisic acid; NCED: 9-cis-epoxycarotenoid dioxygenase; AAO: abscisic aldehyde oxidase; ABA8ox: ABA-8’-hydroxylases. MWG, BB, Cn, Cs, Cs + Spd see Fig 1. Different small letter(s) on top of the bars indicated significant differences (p ≤ 0.05, LSD) among treatments at same sown time.

Chilling stress decreased GA content in seeds of two cultivars, but the inhibitory effect on BB (fold change 1.9–2.6 down-regulated) was higher than that on MGW (fold change 1.3–1.4 down-regulated) compared with Cn treatment. At 1^st^-3^rd^ d of germination, Spd significantly increased the GA content in BB seeds, while no significant difference on GA content was detected between treatments of MGW + Cs and MGW + Cs + Spd (Fig 4A). Chilling stress significantly down-regulated the expressions of GA synthesis genes (*BoGA20ox1*, *BoGA20ox1* and *BoGA3ox*) in both cultivars, and made no effect on *BoGA2ox* expression during seed germination. Furthermore, Spd-induced up-regulated expressions of *BoGA20ox1* and *BoGA3ox* were observed only in BB seeds under chilling stress. By contrast, the transcription level of *BoGA2ox* in both cultivars were not regulated by Spd application under chilling stress (Fig 4B–4F).

### 3.4 Effects of exogenous Spd on ABA content and related-gene expressions during kale seed germination under chilling stress

**Fig 5:**
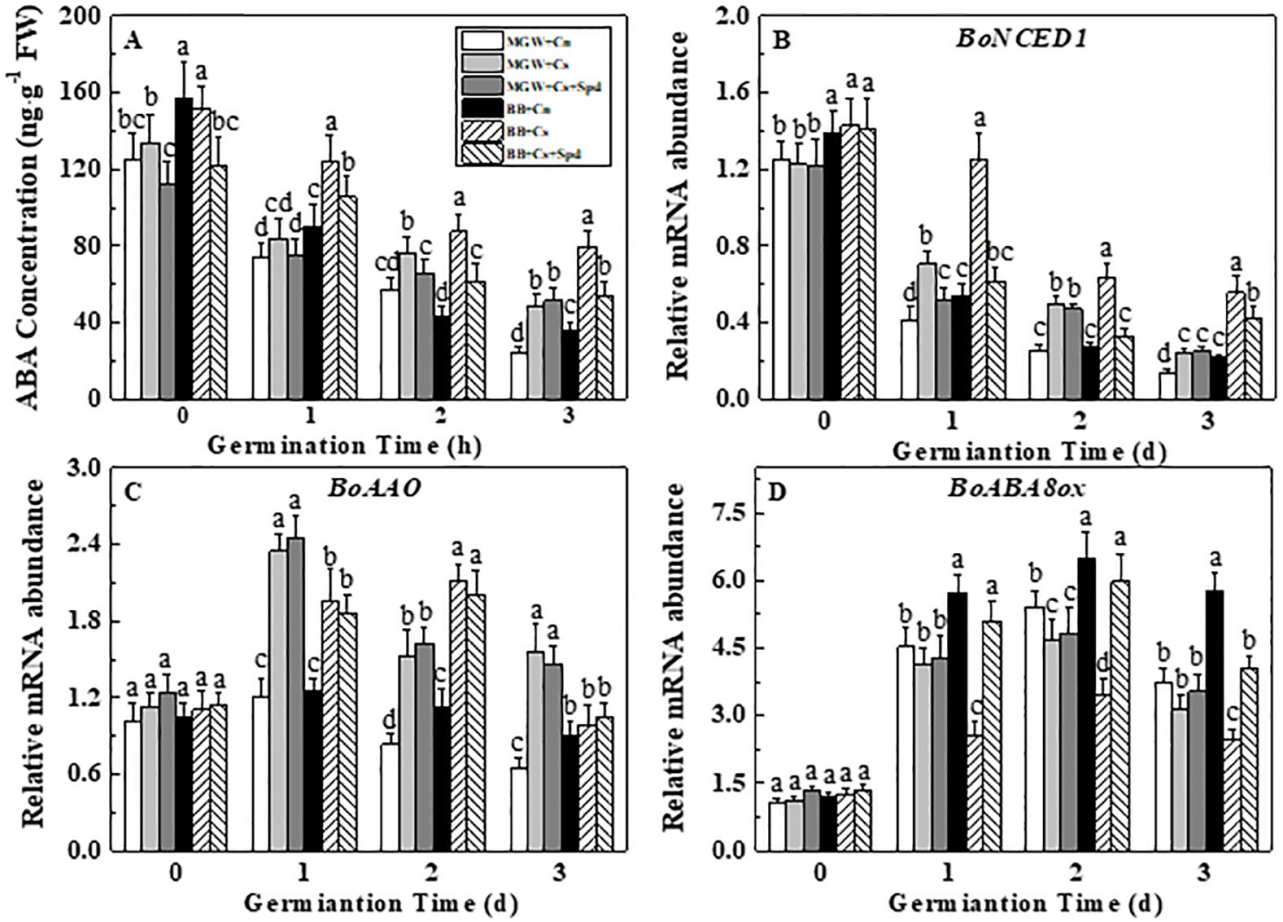
Effects of Spd on ABA content (A) and relative expressions of *BoNCED1* (B), *BoAAO* (C) and *BoABA8ox* (D) of kale seeds during germination time under chilling stress. ABA: abscisic acid; NCED: 9-cis-epoxycarotenoid dioxygenase; AAO: abscisic aldehyde oxidase; ABA8ox: ABA-8’-hydroxylases. MWG, BB, Cn, Cs, Cs + Spd see Fig 1. Different small letter(s) on top of the bars indicated significant differences (p ≤ 0.05, LSD) among treatments at same sown time.

Chilling stress significantly increased ABA content during germination time in BB and MGW seeds. Exogenous Spd significantly lowered ABA content of BB seeds at low temperature, but made no significant effect on ABA content in MGW (Fig 5A). The expressions of *BoNCED1* and *BoAAO* in both cultivars were significantly up-regulated by chilling stress. However, low temperature-inhibited *BoABA8ox* expression was only observed in BB germination process. As expected, Spd application remarkable decreased the expression of *BoNCED1* on 1^st^ and 2^nd^ d of germination of MGW and BB under chilling stress. Moreover, the transcription of *BoABA8ox* was up-regulated by exogenous Spd in BB seeds on 1^st^-3^rd^ d of germination (Fig 5B–5D).

### 3.5 Effects of exogenous Spd on ET content and related-genes expressions during kale seed germination under chilling stress

**Fig 6:**
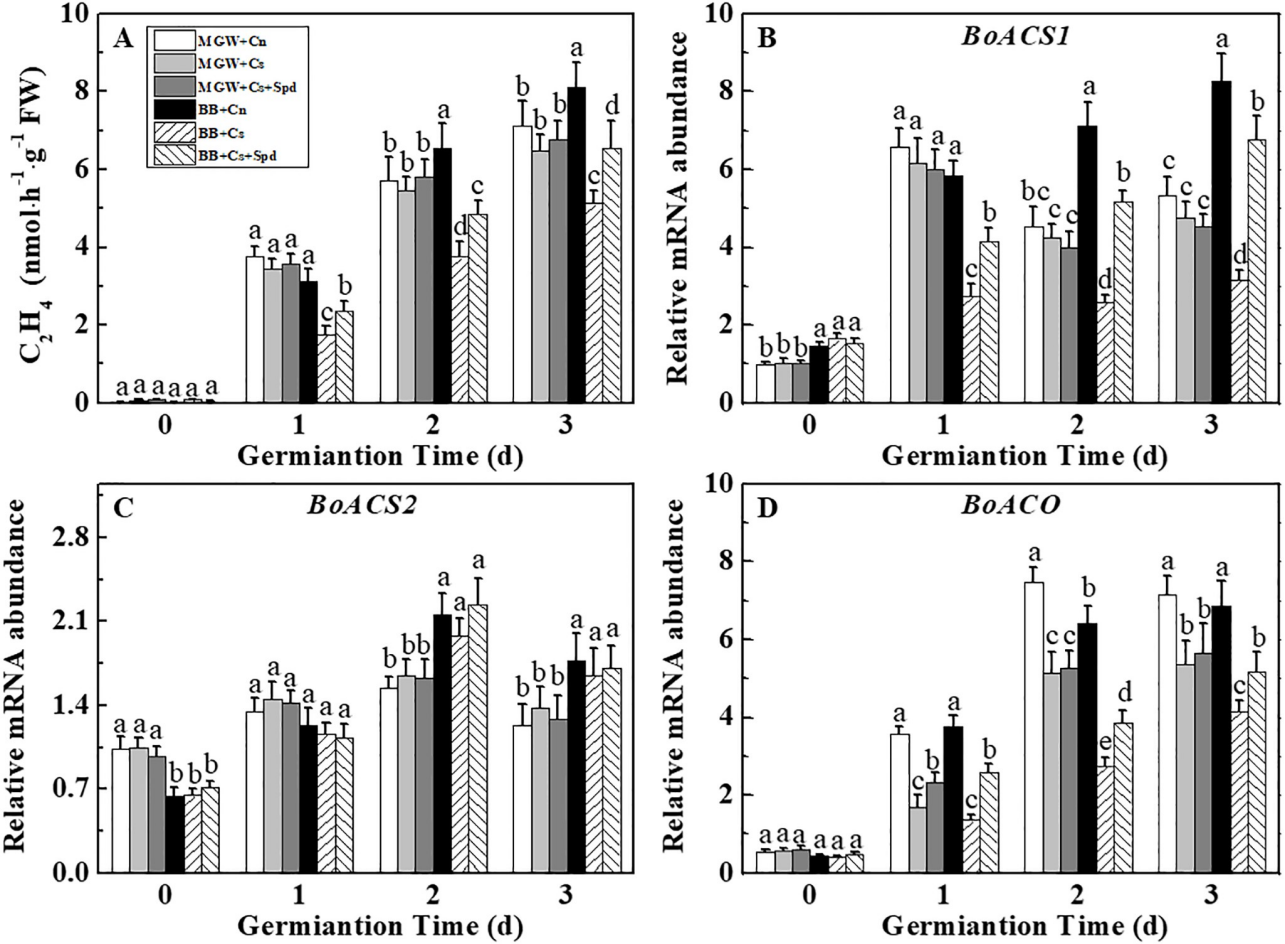
Effects of Spd on ethylene content (A) and relative expressions of *BoACS1* (B), *BoACS2* (C) and *BoACO* (D) of kale seeds during germination time under chilling stress. ACS: 1-Amicocyclopropane-1-carboxillic-acidabscisic acid synthase; ACO:ACC oxidase; AAO: 1-Amicocyclopropane-1-carboxillic-acidabscisic acid oxidase. MWG, BB, Cn, Cs, Cs + Spd see Fig 1. Different small letter(s) on top of the bars indicated significant differences (p ≤ 0.05, LSD) among treatments at same sown time.

The ET content in BB seeds was decreased at 1^st^-3^rd^ d of germination under chilling stress, while it was unaffected by low temperature in MGW. Exogenous Spd significantly increased the ET content in BB cultivar, while exerted no significant effect in MGW cultivar (Fig 6A). Chilling stress significantly decreased the expressions of *BoACS1* and *BoACO* in BB at 1^st^-3^rd^ d of germination. It was worth noting that Spd increased the transcriptional levels of *BoACS1* and *BoACO* under chilling stress (Fig 6B and 6D). Noticeably, chilling stress and Spd had no effect on the expression of *BoACS2* in both cultivars during seed germination (Fig 6C).

### 3.6 Effects of exogenous Spd on contents of H_2_O_2_ and O_2_·^-^ during kale seed germination under chilling stress

**Fig 7:**
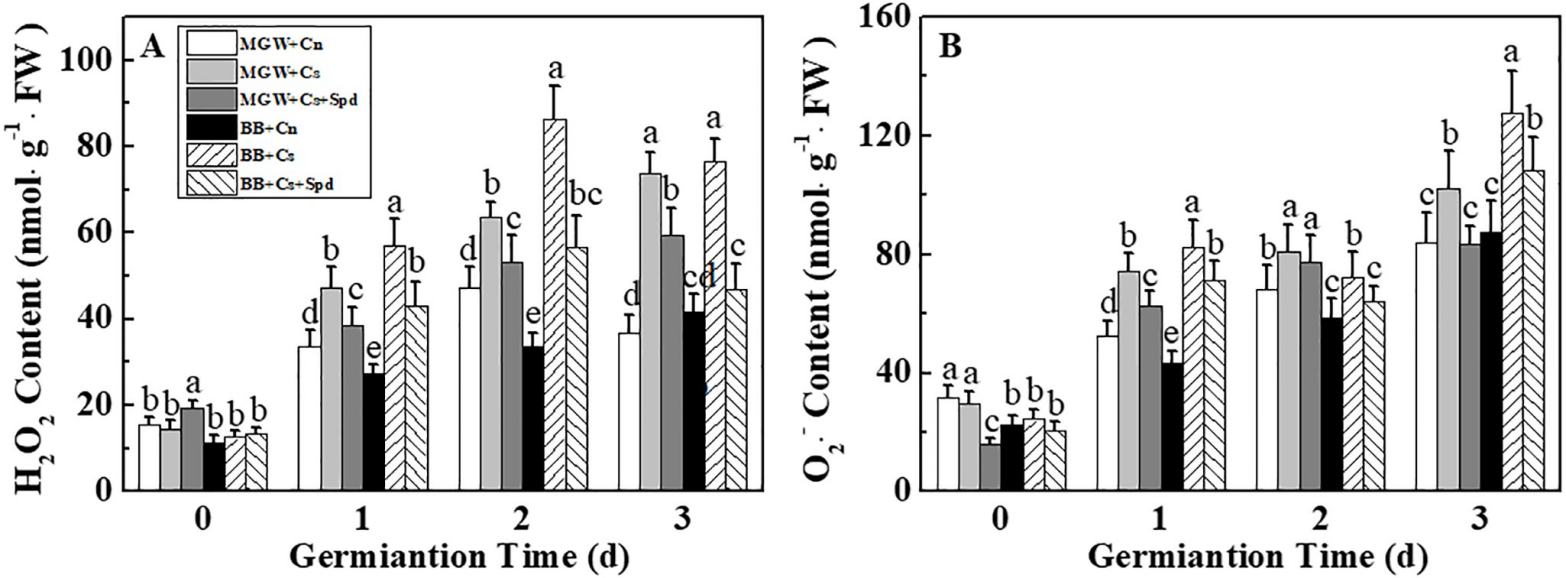
Effects of Spd on H_2_O_2_ (A) and O_2_·^-^ (B) contents of kale seeds during germination time under chilling stress. H_2_O_2_: hydrogen peroxide; O_2_·^-^: superoxide anion; MWG, BB, Cn, Cs, Cs + Spd see Fig 1. Different small letter(s) on top of the bars indicated significant differences (p ≤ 0.05, LSD) among treatments at same sown time.

Chilling stress significantly increased the H_2_O_2_ and O_2_·^-^ levels during the seed germination process in both cultivars (Fig 7). At 1^st^-3^rd^ d of germination, the H_2_O_2_ and O_2_·^-^ contents of MGW + Cs treatment were significantly higher than those in MGW + Cn. In addition, remarkable increased contents of H_2_O_2_ and O_2_·^-^ were observed in BB + Cs seeds compared to BB + Cn seeds. Exogenous Spd significantly reduced the accumulations of H_2_O_2_ and O_2_·^-^ during germination of MGW and BB at 13 °C.

### 3.7 Effects of exogenous Spd on antioxidant enzyme activities and related-gene expressions during kale seed germination under chilling stress

**Fig 8:**
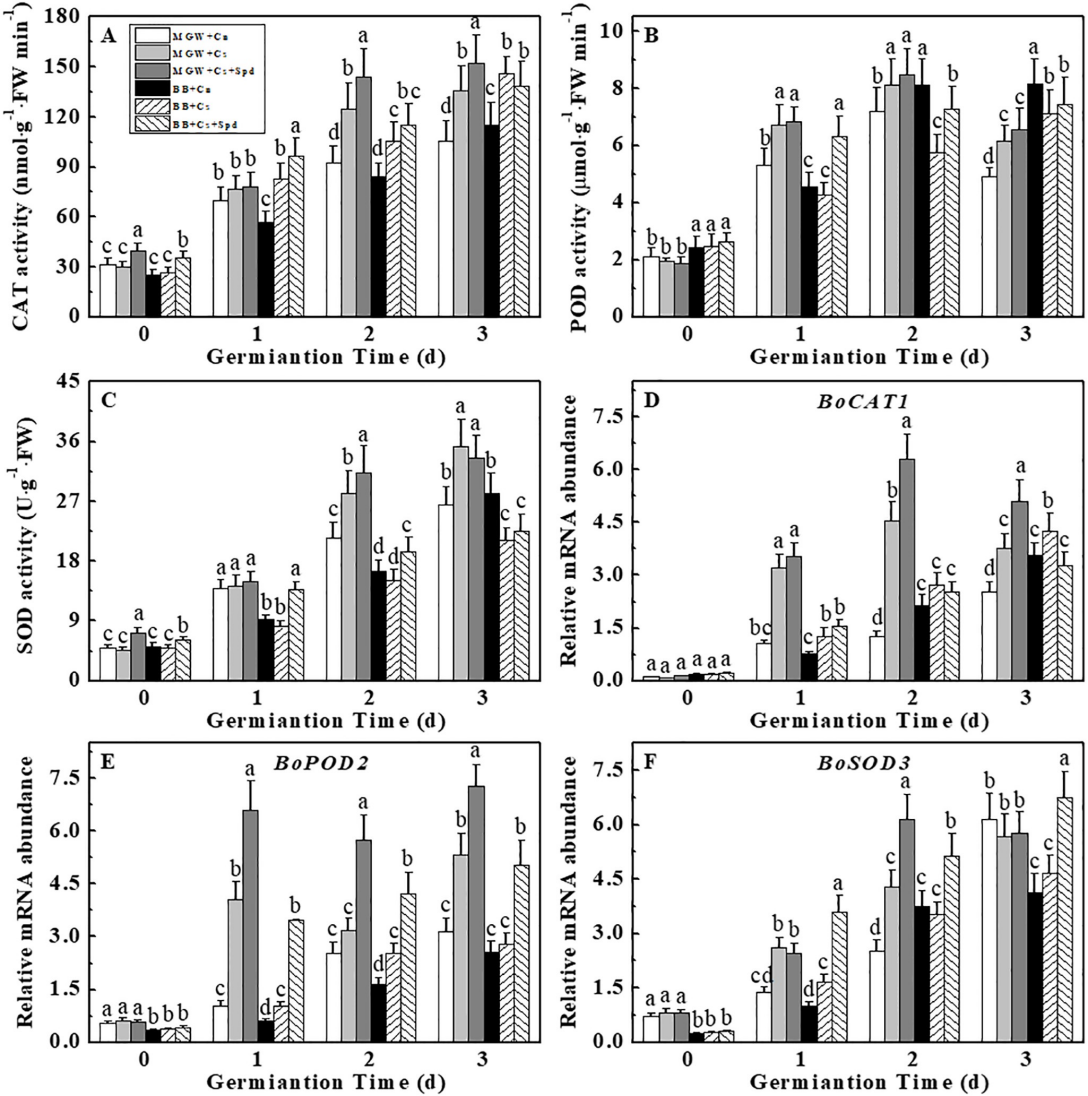
Effect of Spd on activities of CAT (A), POD (B), SOD (C) and metabolism-related genes relative expressions (D-H) of kale seeds during germination time under chilling stress. CAT: catalase; POD: peroxidase; SOD: superoxide dismutase. MWG, BB, Cn, Cs, Cs + Spd see Fig 1. Different small letter(s) on top of the bars indicated significant differences (p ≤ 0.05, LSD) among treatments at same sown time.

Under chilling stress, the CAT, POD and SOD activities in MGW seeds were apparently enhanced, while only a significant increase in CAT activity was observed during seed germination of BB (Fig 8A–8C). Moreover, chilling stress significantly decreased the POD activity in BB seeds on 2^nd^-3^rd^ d of germination. On d 1^st^ and 2^nd^ of germination, exogenous Spd dramatically enhanced the activities of CAT, POD and SOD activities, and up-regulated the expressions of *BoPOD2* and *BoSOD3* in BB. Besides, significant increases in CAT and SOD activities as well as *BoCAT1*, *BoPOD2*, *BoSOD3* expressions were detected in MGW + Cs + Spd compared with MGW + Cs on d 2^nd^ of germination (Fig 8).

## 4 Discussion

Seeds are the foundation of agricultural production. The success of seed germination and the establishment of a normal seedling are vital to plant growth and yield formation of crops [32]. Seed germination is a complicated process controlled jointly by a variety of endogenous and exogenous factors [33]. Poor environmental conditions inhibited crop seed germination and field emergence. Of which, chilling stress is one of the major abiotic stresses that restrict crop seed germination. Our data showed that kale seeds germination and seedling quality on d 7^th^ of germination were significantly inhibited at a low temperature of 13 °C. ‘Pigeon’ (BB) showed a significant lower chilling stress tolerance during seed germination compared with ‘Nagoya’ (MGW).

Spd is a cationic compound ubiquitously distributed in plant, which functions in multiple plant growth and developmental processes including fruit maturation, seed development, seed germination and adaptive responses to abiotic stresses [34]. Notably, Spd plays an important role in plant responses to chilling stress. Exogenous Spd could improve the photosynthetical system function, induce the synthesis of protective active substances, stimulate the antioxidant enzyme activities, and thus enhance the low temperature resistance of tomato [35], eggplant [36], rice [13], cucumber [37] and ryegrass [38] seeds or seedlings. Overexpression of *AtSPDS* increased the expressions of several stress response transcription factors (WRKY, bZIP, rd29A) and improved the chilling stress resistance of *Arabidopsis thaliana* plants [39]. In present study, exogenous Spd significantly increased the GE, GP, GI, VI and seedling qualities of kale cultivar ‘BB’ under chilling stress. Besides, a slight improvement of seed germination ability induced by Spd was also observed in kale cultivar ‘MGW’ under chilling stress. Therefore, Spd was believed to play an important role in physiological metabolism during kale seeds germination at low temperature.

The influences of low temperature upon PAs metabolism differed depending on species, organs and physiological processes. Put was accumulated in wheat, oatmeal and barley seedlings and Spd contents in wheat and barley significantly elevated, while Spm content in wheat was obviously higher than those in barley and oatmeal under cold stress [40]. The endogenous Spd was rapidly accumulated in the cold-resistant cucumber leaves, while that in cold-sensitive cucumber leaves showed no significant change under chilling stress [37]. In this study, chilling stress significantly induced the *BoADC* expression and promoted Put accumulation during the seed germination of the two cultivars. However, MGW seeds showed significant increased Spd content and *BoSPDS* expression under chilling stress; while apparently decreased levels of Spd content and *BoSPDS* expression were observed in BB seeds at low temperature. It was proposed that the response of Spd metabolism to chilling stress might be closely associated with the low temperature resistance of kale seeds during germination. Moreover, our results showed that exogenous Spd could increase endogenous Spd content by up-regulating Spd synthesis related genes expressions. It was suggested that the improvement of kale seed germination under chilling stress by exogenous Spd might be closely related with the metabolism of endogenous Spd.

GA and ABA are major endogenous hormones that control seed dormancy and germination [41]. The maintenance of seed dormancy is dependent on the ABA/GA ratio of seed, and the initiation of seed germination is induced by decreased ABA content and increased GA content [42]. The GA synthetic genes (*GA3ox* and *GA20ox)* were highly expressed at the early stage of seed germination, and promoting the excessive accumulation of GA in seed embryo [18]. GA induces starch degradation in the aleurone layer of rice seed, thus providing substrates and energy for seed germination [43]. During the seed imbibition process, low temperature down-regulated the expressions of *GA3ox1* and *GA3ox2* through regulating transcription factor bHLH and phytochromone interaction factor (PIL5), consequently delaying the seed germination process [19]. In present study, chilling stress significantly lowered the expressions of *BoGA20ox1*, *BoGA20ox1* and *BoGA3ox*, leading to the decrease of GA content during seed germination in both cultivars. Exogenous Spd induced an increase in *BoGA20ox1* and *BoGA3ox* expressions, and alleviated the deficiency of GA content caused by chilling stress in cultivar BB. However, no effect of Spd on GA content was observed in MGW seeds during germination time under chilling stress. The above results suggested that Spd improved seed chilling tolerance might be closely associated with the regulation of GA metabolism at transcriptional levels. However, the influences of Spd upon GA differed depending on cultivars.

ABA plays an important role in plant responses to low temperature stress [27, 44, 45]. Rice carotenoid (ABA-synthesized precursor) deficiency *phs* and *Phs-*RNAi transgenic plant enhanced the cold resistance by decreasing the endogenous ABA level at the seedling growth and reproductive stages [46]. Overexpression of ABA metabolic gene *OsABA8ox1* reduced the endogenous ABA level in rice seedlings, and improved the cold resistance of the transgenic lines [47]. Moreover, the endogenous ABA content in rice seeds showed extremely significant negative correlation with the seed vigor indexes under chilling stress [48]. In present study, chilling stress induced the expressions of *BoNCED1* and *BoAAO*, and increased the endogenous ABA content during kale seed germination. Notably, the increased content of ABA in BB was significantly higher than that in MGW. Exogenous Spd significantly decreased *BoNCED1* expression and increased *BoABA8ox* expression, and thus decreasing ABA content during BB seed germination under chilling stress. Changes in the expression levels of ABA metabolism-related genes might be an important reason for the regulation of Spd on kale seed germination under chilling stress. Consistent with the above results, NCED and ABA8ox were proved be closely involved in the ABA metabolism and plant response to abiotic stress during seed germination [14, 18, 49, 50].

ET plays an important role in plant seed germination and response to chilling stress. ET could negatively regulate the cold resistance of *Arabidopsis thaliana* through the transcriptional regulation of *CBFs* and *ARRs* [51, 52]. However, Catalaa et al. found *Arabidopsis thaliana* ET excess mutant *eto1-3* enhanced the cold resistance by regulating the expression of *CBFs* gene [53]. Similarly, Li et al. reported that exogenous ET could improve the resistance of *Ripe bananas* to cold stress. Thus, it was suggested that the influence of ET upon plant chilling tolerance differed depending on species and physiological processes [54]. In this study, chilling stress significantly decreased ET content of BB seeds through inhibiting the expressions of *BoACS1* and *BoACO*, while the ET content in MGW cultivar was not significantly affected. The difference of chilling tolerance during seed germination between MGW and BB might be related to the response of ET metabolism to chilling stress. The interaction between Spd and ET had been reported in details during plant fruit ripening and seed germination. A significantly enhanced endogenous Spd was detected in *ySAMDC-*overexpression tomato fruits, which simultaneously accumulated 1.5–2.0 folds in ethylene production compared withthe control [55]. Exogenous Spd induced an increase in *ZmACS* expression and ET content, and improved seed germination of sweet corn [27]. Consistently, our study found that Spd application significantly increased *BoACS1* and *BoACO* transcripts, leading to the increase of ET level in Spd-treated seeds. It was suggested that exogenous Spd influenced ET homeostasis by modulating *BoACS1* and *BoACO* expressions during kale seed germination under chilling stress.

The balance of ROS metabolic system in plant is destroyed by chilling stress, thus inducing oxidative stress and membrane lipid peroxidation [56]. Plants can induce antioxidant enzymes, including CAT, SOD, and POD, to counteract the oxidative stress caused by environmental stress [53, 57, 58]. Our results showed that kale seeds exposed to chilling stress showed significant increases in H_2_O_2_ and O_2_·^-^ contents compared to controls. The activities of CAT, SOD and POD, together with the expressions of related-genes, were significantly up-regulated in MGW seeds at low temperature. However, for the cold-sensitive cultivar BB, the POD activity was significantly decreased by chilling stress treatment, illustrating the different responses between cultivars. Additionally, exogenous Spd increased the expressions of *BoCAT1*, *BoSOD3* and corresponding enzyme activities, thus decreasing the accumulations of H_2_O_2_ and O_2_·^-^ of BB seeds under chilling stress. While the POD activity and related-genes expressions were not significantly affected by exogenous Spd in both cultivars, suggesting that CAT and SOD might be the key enzymes in the regulation of exogenous Spd upon antioxidant enzyme system during kale seed germination at low temperature. Similarly, Diao et al. reported that Spd application significantly increased the activities of CAT and POD and improved the cold resistance of tomato seedlings [59]. Besides, exogenous Spd could increase the antioxidant enzyme activities, maintain the cell membrane stability, and thus promoting the seed germination of rice at low temperature stress [13]. The above studies suggested that Spd could participate in the plant cold resistance establishment through osmotic adjustment and ROS scavenging.

## 5. Conclusions

In general, our data confirmed that Spd metabolism played an important role in kale seed germination under chilling stress. During seed germination, MGB was cold-tolerant, while BB was cold-sensitive under chilling stress. Exogenous Spd could improve the seed germination and seedlings characteristics of kale by alleviating the over-accumulation of ROS content and increasing antioxidant enzyme activities. In addition, the effect of exogenous Spd on the metabolism of endogenous Spd, GA, ABA and ET might be an important reason for promoting the seed vigor of kale seeds at low temperature. In ongoing research, we aim to examine the underlying genetic mechanisms using CRISPR gene editing, and we hope that the precise mechanisms underlying the positive effect of Spd on kale seed chilling tolerance will be uncovered in the near future.

## Supporting information

S1 File (XLSX)

S1 Table Primers used in Real-Time Quantitative PCR. (DOCX)
