# Supplementary material for: Spermidine enhances chilling tolerance of kale seeds by modulating ROS and phytohormone metabolism
Source: PLoS One. 2023 Aug 3;18(8):e0289563. doi: 10.1371/journal.pone.0289563 (PMC10399780; doi:10.1371/journal.pone.0289563)
Supplement: S1 Table — (DOCX) [file pone.0289563.s002.docx]

Table S1. Primers used in Real-Time Quantitative PCR.

| Gene name | Primer sequence |
| --- | --- |
| *actin* | F: 5´- GGAAGGACTTGTACGGTAACATTG -3´  R: 5´- TGGACCTGCCTCATCATACTCA -3´ |
| *BoADC* | F: 5´- CAGTCCGCGTTCGATTTC -3´  R: 5´- CCGGGTACACTCCTTGGTAA -3´ |
| *BoSAMDC1* | F: 5´- AGGAACGGTGATGTATCAGAGG -3´  R: 5´- GATCTCGGAGAACCGCAATA -3´ |
| *BoSAMDC3* | F: 5´- GGTCCCAGCTTGACGAGATA -3´  R: 5´- CGTTGGAGAGAGAGGAAACG -3´ |
| *BoSPDS* | F: 5´- CCTGTGAACCCAATTGATGA -3´  R: 5´- CGTTGTAAAACTTTAAGGGTCCA -3´ |
| *BoSPMS* | F: 5´- TGTTCTGGTTGTTGGTGGAG -3´  R: 5´- TCAACAGAGCTATGGCGAGA -3´ |
| *BoGA20ox1* | F: 5´- TTGACGTTCCCCTCATCG -3´  R: 5´- CTAAAGTGGCGGAGGGAGA -3´ |
| *Bo GA20ox2* | F: 5´- TCACGAACCGTTCAGGATT -3´  R: 5´- CCCAAATTGCTCGAATTCTT -3´ |
| *Bo GA3ox* | F: 5´- CCAAGCCCCTCTCATAGACC -3´  R: 5´- GCTTCTGAGACCAAGAACGAA -3´ |
| *Bo GA2ox* | F: 5´- TGACGTTGGGTGGATTGAG -3´  R: 5´- TTTAGGGGTGGAGAGCTGAG -3´ |
| *BoNCED1* | F: 5´- AGATGATGGCCGGAAACTC -3´  R: 5´- ATTATCCCCAATCGCGAAA -3´ |
| *BoAAO* | F: 5´- CGGCGGTAATCTAGTGATGG -3´  R: 5´- AGAAGCGTGGCGATATCAGA -3´ |
| *BoABA8ox* | F: 5´- CTCGTCCTCCGTGCTTTC -3´  R: 5´- TTGATTCGATGTCGGGAAC -3´ |
| *BoACS1* | F: 5´- CTTGCACTCGAAGAAGCCTAC -3´  R: 5´- TGAGAACTCCTTTGACGTTTAGG -3´ |
| *BoACS2* | F: 5´- TTCAGAACAACAATGCTAAGAAGC -3´  R: 5´- GTCGGAAACTCAGTCGGAGA -3´ |
| *BoACO* | F: 5´- TCATGGAACAAAAGGTCCAAC -3´  R: 5´- TGGCTTAGGACAAGCTGGAT -3´ |
| *BoCAT1* | F: 5´- ATCCTCGTGGTTTTGCTGTC -3´  R: 5´- TGCCAACAAGATCAAAGTTCC -3´ |
| *BoPOD2* | F: 5´- GGTGGTTTCTTGCGCTGA -3´  R: 5´- CCATGGTCCGTTGATCACTA -3´ |
| *BoSOD3* | F: 5´- TGGTGATCCTGATGACCTTG -3´  R: 5´- CCTGCGTTTCCTGTTGATTT -3´ |
